# Letters to the Editor

**DOI:** 10.1080/13577149877966

**Published:** 1998-12


*Sarcoma* (1998) **2**, 193–196

CARFAX

### LETTERS TO THE EDITOR

*Sir*: Many thanks for asking me to comment on the Dutch consensus document on 'Diagnosis and Treatment of Soft Tissue Tumours'.

The Dutch consensus document on soft tissue sarcoma (STS) is well done, comprehensible and apart from minor remarks it could be basically accepted as a standard.

Nevertheless, the document gives the impression that agreement exists among specialists in the treatment of STS, and that by simply following these guidelines, everyone is allowed to treat these lesions. In practice this is far from true.

In our major Italian cancer centre, and it happens in many other European institutions, 60% of STS are seen for rescue treatment after inadequate therapies performed elsewhere. After important progress in surgical culture and procedures, the likelihood of achieving a good local control improved, but only in major institutions.

Results of treatment remain very poor. Surgeons and radiotherapists insist on stressing their important role while up to 50% of patients with high-grade tumours die from metastatic disease waiting for a missing effective medical treatment. In these circumstances nothing up to now can be standardized or imposed.

Surgery is also not optimal even where skill and facilities are best, but the overall survival for metastatic spread discourages us from more heavy, mutilating or dangerous procedures. The document discusses the possible dangerous impact of the unexpected finding of sarcoma, typically after minor operations performed in non-oncological departments. Despite expectations, these patients have, in our series, a better prognosis (data reported at the ECCO Conference, Hamburg 1997). This fact is probably due to a favourable selection of patients presenting with small superficial lesions with a slow growing pattern.

Radiotherapy (RT) is accepted as a necessary procedure complementary to surgery, but it is common experience that its effect is proportional to the quantity of tumour tissue left behind; the treatment is acceptable if only a microscopic residual is left after an adequate macroscopic complete resection. It is ineffective if macroscopic residual tumour is left, even if minimal. It should be stressed, and it is not clear in the document, that RT is not a remedy for incomplete or improvable surgery. Again the timing of RT is not yet standardized. Many centres have a satisfactory experience with pre-operative RT, and in our Institute we are performing a new protocol with pre-operative RT for retroperitoneal tumours.

Chemotherapy is a most confused topic. Reading the literature, adjuvant chemotherapy is not indicated outside clinical trials. The meta-analysis stimulates ethical questions and in the near future other investigators will probably state that adjuvant chemotherapy is mandatory in selected cases.

A minor remark is that the term of 'compartment', so frequently adopted since the early 1980s, is now obsolete. In fact the only useful compartment that determines the quality of surgery is the muscular fascia. The definition of intramuscular or extramuscular lesion should be preferred.

In conclusion sarcomas are rare, the data confused and physicians should not have the privilege to treat patients by their own feeling. It is more ethical for any eligible patient to be entered into a clinical trial, and that for any simple or complicated case of STS, or where it is suspected, the physician not familiar with this disease should contact a specific centre.

A. Azzarelli
*Istituto Nazionale Tumori*
*Milano, Italy*

*Sir*: Many thanks for asking me to comment on the Dutch nationwide accepted consensus document on 'Diagnosis and Treatment of Soft Tissue Tumours'.

There is no doubt that the mechanism by which this consensus document was drawn up has in itself been very therapeutic. It appears to have been democratically derived after a total of nine meetings and clearly much thought and effort has gone into its production.

It is a very prescriptive document, defining what should be done in virtually every circumstance drawing on standard principles of sarcoma care. I have no doubt that the clinicians who attended the meetings and who were involved in the production of these guidelines were all better informed and better equipped to deal with the challenges posed by soft tissue sarcomas at the completion of the exercise.

But there are three problems posed by documents such as this:

1. The guidelines are very precise and allow for little latitude. Non-adherence to these guidelines may now be unacceptable—both

1357-714X/98/030193-04 $9.00 © 1998 Carfax Publishing Ltd clinically and medico legally. Any aberration from the defined pathway may lay that clinician open to claims of an unacceptable or improper standard of care for that patient. This may be the case but equally it does not allow for latitude of innovation, particularly for the experienced clinician. For the occasional surgeon, however, these guidelines represent a minimum standard of care from which he or she varies at his/her peril.

2. These guidelines make turgid reading. How many clinicians who were not actively involved in drawing up the original document will have read them or even be aware of them. How many will go and look it up when and if they get a patient with a soft tissue sarcoma? For guidelines to be effective there needs to be a national consensus of not only how the patient should be treated but also by whom the patient should be treated. Should all patients be treated at a national centre or is there still room for the 'occasional' surgeon in this field of oncology. What are the outcomes, does it matter?—I believe that these are still questions without complete answers.
3. Any document such as this must have an acknowledged time limit on it such that the guidelines are reviewed and modified appropriately in light of changing findings and fashions.

How might guidelines such as this be used in the United Kingdom?

It is an unfortunate fact that in the United Kingdom soft tissue sarcomas continue to be managed by a variety of surgeons using a whole host of treatments many of which could not be considered optimal in light of current knowledge. There is acceptance that treatment and outcomes tend to be better if patients are treated in specialist centres for all forms of cancer but the data confirming this is simply not available for soft tissue sarcomas.

Introduction of a document such as this would help to standardize treatment in the bigger centres (where treatment will already follow accepted guidelines) but will probably not influence in the slightest the occasional surgeon who is unlikely to read let alone acknowledge a document such as this. He will rightly insist that there are no data showing that treatment in a specialist centre is better for the individual. Until this is forthcoming I firmly believe that the most that can be done is to set a minimum standard of care and to widely circulate this in a brief and succinct document (see Table 1).

National guidelines should indeed be reached by consensus but should ideally follow the recognition of the strengths and weaknesses of a country's current outcomes in an attempt to improve these.

R. J. Grimer
*Birmingham Orthopaedic Oncology*
*Birmingham, UK.*

**Table 1**. *Document describing minimum standard of care in treatment of soft tissue sarcomas*

### MANAGEMENT GUIDELINES FOR BONE AND SOFT TISSUE SARCOMAS.

### Introduction

Bone and soft tissue sarcomas are a rare and heterogeneous group of tumours. Many clinicians will never see a case during their professional career. Their recognition is important however, because timely investigation and treatment can result in cure. Their management requires close collaboration between designated specialists in a multidisciplinary team and early referral to such a regional or supra-regional service will lead to the best clinical and cost effective care.

There guidelines have been produced in line with current accepted practice not only in this country but within Europe and the United States. They stem from consensus conferences in these countries and from published guidelines which are circulated to all interested parties in these countries. There guidelines will be submitted for agreement to the Royal College of Surgeons, The British Orthopaedic Association, The Medical Research Council.

### Presentation

Primary bone tumours can occur at any age. Whereas oteosarcoma and Ewing's sarcoma have their peak incidence in adolescence and early adulthood, chondrosarcoma and osteosarcomas secondary to radiation damage and Paget's disease increase in frequency with age. Pain is the commonest presenting symptoms in patients with primary bone tumours. Unfortunately, this is a common complaint after minor trauma and sporting injuries, and a high index of suspicion is required. Features suggestive of bony malignancy are:

- night pain
- non-mechanical pain (ie. continuous pain not aggravated by exercise)
- swelling that is not associated with a joint

The changes seen on plain radiographs can be subtle, such as periosteal reaction, bone destruction, new bone formation (calcification) and soft tissue swelling. These findings are not specific of a bone tumour but are suggestive and always warrant further investigation.

Soft tissue sarcomas increase in frequency with age. Some (particularly in younger patients) may be associated with familial syndromes such as neurofibromatosis and Li-Fraumeni syndrome. They usually present as a painless mass. Features of a soft tissue lump suggestive of malignancy are:

- size $>5$ cm diameter
- enlarging
- painful
- deep to fascia
- solid mass (not cystic)
- recurrence at site of previous excision (whatever the previous histology)

Any such lesions should immediately be referred to a specialist sarcoma service.

### Investigations

The biopsy should ideally be undertaken by the specialist surgeon who will be responsible for the definitive operation because the biopsy tract will need to be resected at that procedure. An excision biopsy or 'shell-out' is never appropriate because sarcomas typically form a pseudocapsule that is not a barrier to spread. The increasing use of cytogenetic analysis in diagnosis and monitoring of treatment requires fresh tissue from the initial biopsy.

When a sarcoma is suspected or demonstrated, staging investigations should be performed by the specialist team before biopsy, because haematoma, oedema and scar tissue reduce the yield of diagnostic information and delineation of the tumour extent, particularly in relation to neurovascular structures. Films of suspected sarcomas should be reviewed by Radiologists trained and experienced in this work. The following are required before biopsy to define the primary tumour:

- high definition plain radiographs in two planes
- C.T. or preferably M.R.I scan of the tumour

The following are required before definitive surgery to exclude metastases:

- CXR and C.T scan of thorax
- bone scan (for primary bone tumours)
- FBC, U + E, LFT, LDH
- in selected cases, bone marrow biopsy

Close co-operation is required between the radiologists, surgeon, pathologist and oncologists. Sarcomas are not only a heterogeneous group of tumours but a wide range of appearances can occur within individual tumours. Special training and membership of national bone or soft tissue sarcoma pathology panels is required to gain experience in the interpretation of these rare tumours. In order to be able to comment on grade, necrosis, mitotic rate and adequacy of the margins, the pathologist must be able to orientate the specimen accurately before tissue retraction occurs. Trojani grading should be used for soft tissue sarcomas. Pathology review by a sarcoma panel is obligatory for entry into clinical trials and leads to revision of the diagnosis in 10–30% of cases.

### Definitive Surgery

Surgery for primary bone tumours and most soft tissue sarcomas should only be undertaken by appropriately trained surgical oncologists. This will usually imply an orthopaedic oncologist for bone tumours or a surgeon working in a recognised sarcoma unit with appropriate training and experience. It is essential they work within a multidisciplinary team with appropriate support facilities. There is no place for the "occasional operator" for these tumours.

Surgery is defined as:-

*Incomplete*—when any macroscopic tumour is left in situ. This requires re-excision.
*Marginal*—when the pseudocapsule is visible or any clearance margin is $< 1$ cm without fascia
*Wide* - when there is a cuff of normal tissue at least 1 cm laterally (or a fascial plane) and 3 cm longitudinally
*Radical*—when the entire muscular compartment
Contaminated—when rupture of the pseudocapsule results in tumour spillage

### Adjuvant Treatment

For primary bone tumours, pre and post operative chemotherapy should be given in accordance with the current MRC/EOI protocols. Referral to a regional oncology unit is essential for this.

For soft tissue sarcomas, the role of adjuvant chemotherapy is uncertain. Patients with high grade tumours should be considered for enrolment into the current EORTC soft tissue sarcoma study. This requires an early decision regarding chemotherapy and all cases should be discussed at a multidisciplinary meeting following definitive histology of the resected tumour. Adjuvant radiotherapy is required for most soft tissue sarcomas and close links between the operating surgeon, surgical unit and the local radiotherapist are essential.

### Follow up and Recurrence

Patients can present with local recurrence of any sarcoma many years after primary surgery. They should be promptly referred back to the specialist sarcoma team for consideration of definitive surgery.

Some patients will have metastases at presentation and many others will develop them at a later date. Some of these, particularly pulmonary metastases, can be treated by surgery. For this reason, sarcoma patients should be followed up by the specialist sarcoma team, with regular CXRs. Widespread metastases are incurable, but can be palliated by chemotherapy in a proportion of case. Treatment should be given by a medical oncologist in the specialist team using current protocols of the MRC and EORTC groups.

**PROMPT REFERRAL OF ANY SUSPECTED MUSCULOSKELETAL MALIGNANCY TO A REGIONAL/ SUPRAREGIONAL CENTRE IS STRONGLY RECOMMENDED.**

